# When spermatogenesis meets human aging and elevated body mass

**DOI:** 10.1093/lifemedi/lnac022

**Published:** 2022-07-09

**Authors:** Xiaoyan Wang, Bradley R Cairns, Jingtao Guo

**Affiliations:** State Key Laboratory of Stem Cell and Reproductive Biology, Institute of Zoology, Chinese Academy of Sciences, Beijing 100101, China; Beijing Institute for Stem Cell and Regenerative Medicine, Beijing 100101, China; Howard Hughes Medical Institute, Department of Oncological Sciences and Huntsman Cancer Institute, University of Utah School of Medicine, Salt Lake City, UT 84112, USA; State Key Laboratory of Stem Cell and Reproductive Biology, Institute of Zoology, Chinese Academy of Sciences, Beijing 100101, China; Beijing Institute for Stem Cell and Regenerative Medicine, Beijing 100101, China; University of Chinese Academy of Sciences, Beijing 100049, China

Aging is a biological process that takes place naturally in all living organisms, and affects the physiology and functionality of almost all organ and tissue types, including the reproductive tracts. The ultimate impact of aging in reproduction differs in women and men: while women stop menstruation and ovulation at the age of around 50, men can maintain the ability to produce sperm for the vast majority of their lifetime. Diverse developmental trajectories of germline stem cells may lead to this difference in genders. Specifically, spermatogonial stem cells (SSCs) in the testis remain relatively constant all life, while germline stem cells analog SSCs in men unlikely exist in the adult ovary of women. Intriguingly, although many older men display declined fertility, certain individuals have relatively normal fertility status, as measured by sperm count. The factors responsible for such heterogeneity may be due to differences in individual genetic background, and diverse environmental exposures. Regardless, understanding how aging affects men’s fertility requires delineation of the detailed underlying molecular mechanisms as well as examination of the influence of potential associated factors such as lifestyle, which may provide new avenue to identify novel therapeutic targets or develop effective intervention options to improve the reproductive health of men.

The integrity of testis physiology is critical for successful spermatogenesis, and germline cells are supported and guided by testicular somatic cells, including Sertoli cells (which nurture and support germ cells), Leydig cells (which produce testosterone), and the testicular peritubular cells (TPCs; which contribute to the basement membrane). Aging can lead to several physiological alternations in the testis, such as fibrosis, deposition of extracellular matrix, and increased basement membrane thickness [1]. Understanding the molecular mechanism underlying those changes is of great clinical and scientific significance. Leveraging the unique access to human testis samples through rapid autopsy, recent work took advantage of single cell transcriptomic profiling approach and examined changes in the key components in the human testis during human aging [2], revealing multiple altered pathways. These included elevated inflammation as a globally shared pattern among testicular somatic cells, dysregulated metabolic signaling in Sertoli cells, altered hedgehog signaling and testosterone production in Leydig cells, and improper cell growth in the TPCs. Moreover, while young fertile men share relatively consistent molecular signatures, different degrees of the molecular changes listed above are observed in older men, prompting further investigation into the consequences of those changes.

**Figure 1.**
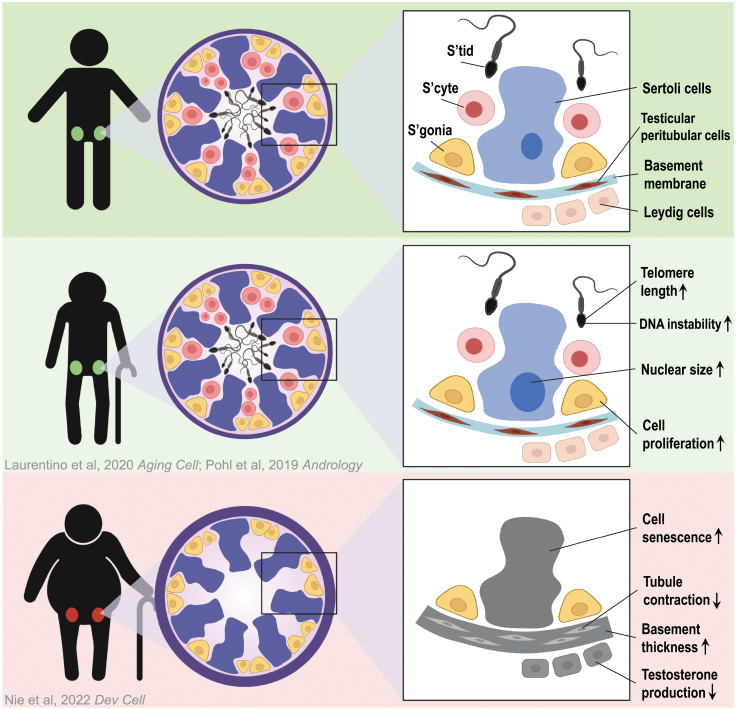
Model illustrating the impaired spermatogenesis and fertility of aging men with normal or high BMI [2, 4, 5].

How does this aging-related molecular dysregulation of testicular somatic cells impact spermatogenesis and fertility? The outcome seems to depend on the extent of the molecular disruption. Older men with relatively modest molecular alternations display complete spermatogenesis with normal fertility, while those with severe alternations exhibit spermatogenic defects. Human spermatogenesis starts with the differentiation of SSCs into differentiating spermatogonia, which enter into meiosis to give rise to spermatocytes, culminating in the formation of haploid gametes—sperm. Interestingly, based on histological examination and transcriptomic analysis, older men with spermatogenic defects typically have regressed germ cell differentiation but relatively intact SSCs, which display normal SSC transcriptomic signatures, indicating that SSCs are less responsive to changes caused by aging (Fig. 1). One possible interpretation is the “survivor bias,” i.e. SSC diminishment via apoptosis might be too swift so that only viable SSCs with “normal” transcriptomes can be captured. However, the SSCs in older men display high variations in colonizing efficiency when transplanted into the testes of immune-comprised mice, suggesting additional layers of aging-related regulation exist in these SSCs. For instance, older men may accumulate more genetic mutations in their SSCs, in which a small proportion can result in increased proliferation and the ability to outcompete their peers—a phenomenon termed spermatogonia selfish selection [3]. Another possible explanation is that SSCs in older men may experience changes in their epigenome (such as DNA methylation, histone modifications, and small RNA repertoire) in response to aging, which could also impact their function [6, 7]. It has been widely reported that epigenetic alternations take place in the gametes, which can be transmitted to the offspring and influence their health [8]. Here, it still remains elusive whether those epigenetic alternations take place prior to spermatogenesis—within the germline stem cells—or instead emerge during germ cell differentiation in the advanced germ cells. If these epigenetic changes indeed take place in SSCs, they may also impact SSC development and maintenance as spermatogenesis progresses.

When one examines the genomics analyses alongside the morphological results, it becomes obvious that the testicular somatic cells display much more pronounced changes in transcription than the germ cells during human testis aging. Notably, Sertoli cell numbers declined significantly in older men, accompanied with diminished metabolic function. As another key testicular somatic cell type, Leydig cells display age-related dysregulation in testosterone production as well. Therefore, somewhat surprisingly, somatic cell aging appears to be the primary driver of testis aging that leads to abnormal fertility in certain older men. This provides an opportunity—as it raises the possibility of targeting the somatic components of the aging testis to delay the decline in ­aging-associated fertility in young men, or to restore fertility in older men. Had the age-related dysregulation been focused on the germ cells and genome integrity, such approaches may not be possible or reversible. However, the discovery of particular disrupted signaling pathways in somatic cells may serve as a potential resource for therapeutic targets to improve reproductive health, which may require further mechanistic investigation involving animal models. For example, previous studies already revealed the critical role of the p53-p21 signaling pathway in regulating cellular senescence. Elevated p53-p21 signaling was also observed in the TPCs of the older men; however, whether this is causative or correlative still awaits further exploration. An inducible p21-Cre mouse model which was recently developed to monitor p21+ senescent cells, which could serve as a useful model for further studies [9].

Aging is associated with many other concurrent factors, such as elevated body mass index (BMI), imbalanced nutrition, decline in exercise, and diabetes. A key question is whether and how these concurrent factors contribute to the heterogeneity of fertility outcomes in older men? Notably, BMI has recently been identified as a critical factor that is positively correlated with reduced fertility and more pronounced dysregulated molecular phenotypes in the somatic cells as compared to low BMI counterparts in the same age range. Intriguingly, BMI appears to have little to no effect on young testis (Fig. 1). What remains unknown is how and when obesity and other correlated attributes play a role in the testis. Systemic inflammation has been observed in individuals with high BMI, which could impact testis through the circulation system. However, we cannot rule out the possibility that obesity may impact the testis and spermatogenesis directly through an unknown mechanism. It is also worth noting that this correlation only exists in older men but not young men, which display no strong/obvious changes in testis molecular signature with high BMI, suggesting that the impact of elevated BMI may be exacerbated in the background of aging. Why are older men more prone to be impacted by obesity than young men? One possible explanation is “robustness”—that older men have a reduced buffering system resulting in declined ability to recover when challenged. However, the challenges may not be limited to obesity. Obesity is also a complex trait associated with multiple additional factors such as elevated risk of cardiovascular disease and metabolic disorders, raising questions regarding roles for those factors. To properly address those questions, a more comprehensive study involving a large patient cohort is needed. The current study in Nie et al. provides sufficient preliminary results to motivate a large-scale investigation [2]. Aging is a process that cannot be reversed in totality; however, it will be of high interest to study whether the molecular and physiological phenotype can be reversed when the external factors are no longer present. If so, at what developmental stage would be reversal be most pronounced? This knowledge would provide highly useful guidelines for healthy lifestyle. Here, the current study compared young (17–25 years) to older (>60 years) males. The availability of additional age-stratified samples between 25–60 years of age will provide the opportunity to determine when and in which cell type molecular dysregulation first occurs in normal versus high BMI individuals. This will be important for determining cause versus consequence, and will help focus future studies on possible reversibility. Thus, the current study prompts extensive follow-up functional studies, and highlights the great potential of intervening in specific pathways or lifestyle changes in managing male reproductive aging.
